# The Hepatoprotective Effect of Jaboticaba Peel Powder in a Rat Model of Type 2 Diabetes Mellitus Involves the Modulation of Thiol/Disulfide Redox State through the Upregulation of Glutathione Synthesis

**DOI:** 10.1155/2018/9794629

**Published:** 2018-08-01

**Authors:** Andréia Quatrin, Lisiane Conte, Dariane Trivisiol da Silva, Cassieli Gehlen Figueiredo, Sabrina Somacal, Miguel Roehrs, Cibele Ferreira Teixeira, Fernanda Barbisan, Paula Rossini Augusti, Mário Roberto Maróstica Júnior, Ivana Beatrice Mânica da Cruz, Tatiana Emanuelli

**Affiliations:** ^1^Graduate Program Food Science and Technology, Centre of Rural Sciences, Federal University of Santa Maria, 97105-900 Santa Maria, RS, Brazil; ^2^Integrated Centre for Laboratory Analysis Development (NIDAL), Department of Food Technology and Science, Centre of Rural Sciences, Federal University of Santa Maria, 97105-900 Santa Maria, RS, Brazil; ^3^Graduate Program on Pharmacology, Centre of Health Sciences, Federal University of Santa Maria, 97105-900 Santa Maria, RS, Brazil; ^4^Biogenomic Laboratory, Department of Morphology, Centre of Health Sciences, Federal University of Santa Maria, Santa Maria, RS, Brazil; ^5^Department of Food Science, Federal University of Rio Grande do Sul, 91501-970 Porto Alegre, RS, Brazil; ^6^School of Food Engineering, University of Campinas, 13083-862 Campinas, SP, Brazil

## Abstract

Jaboticaba peel powder (JPP) is rich in bioactive compounds, mainly soluble and insoluble polyphenols with great antioxidant properties. The aim of this study is to evaluate the effects of JPP supplementation on the oxidative stress and hepatic damage in a rat model of type 2 diabetes mellitus (T2DM). Diabetic rats received vehicle or JPP at 2.7 (JPP-I), 5.4 (JPP-II), or 10.8 (JPP-III) g/L in drinking water during 8 weeks. JPP-III attenuated hyperglycaemia and dyslipidemia increased by 86% the liver content of nonprotein thiol groups and by 90% the GSH/GSSG ratio by activating glutathione synthesis. Accordingly, JPP supplementation prevented the loss of activity of the sulfhydryl-dependent enzyme *δ*-aminolaevulinic acid dehydratase and attenuated hepatic injury assessed by the reduction of serum aspartate aminotransferase activity and liver hypertrophy. Our results support that JPP supplementation to T2DM rats decreases hepatic damage most likely by increasing glutathione synthesis and modulating the thiol/disulfide redox balance.

## 1. Introduction

Diabetes is a major public health problem that affected about 387 million persons worldwide in 2014. This figure is expected to reach 592 million by 2035 [1]. Type 2 diabetes (T2DM) is characterised by hyperglycaemia and a variable degree of insulin resistance in tissues, as well as, disturbances in the metabolism of lipids, protein, and carbohydrates [2]. Chronic hyperglycaemia contributes to oxidative stress, which has been hypothesized as a key component in the development of diabetic macrovascular complications [3] and hepatic damage [4].

Liver is a crucial organ for both lipid and carbohydrate metabolism [4]. Lipid and protein oxidation as well as the depletion of endogenous enzymatic and nonenzymatic antioxidants have been shown to contribute to liver membrane damage in diabetic models [5]. The tripeptide glutathione is a major nonenzymatic antioxidant that plays a key role in the maintenance of hepatic redox balance and protection against diabetes-induced liver damage [6, 7]. In addition, the accumulation of free fatty acids (FFA) in T2DM increases the hepatic production of very low-density lipoprotein (VLDL) and impairs insulin signalling [8]. Such events contribute to the nonalcoholic fatty liver disease [4] and dyslipidemia, which increase the risk of cardiovascular disease in diabetic patients [9].

Oral hypoglycaemic drugs and insulin are used to treat T2DM but are not effective against the development of macrovascular complications and usually cause side effects, such as hypoglycaemia, weight gain, and gastrointestinal intolerance [10]. Thus, nutritional strategies to attenuate diabetic complications would be of great benefit.

Jaboticaba tree (*Myrciaria jaboticaba* (Vell.) Berg.) is native from Brazil, belonging to the Myrtaceae family [11]. Jaboticaba fruit is very tasty and can be consumed fresh or as juice, liqueurs, and jams [11]. It has attracted attention due to the high content of phenolic compounds, mainly anthocyanins and ellagitannins that are mostly found in the fruit peel [11, 12]. Jaboticaba also contains insoluble phenolic compounds that are covalently bound to cell wall structural components such as fibre and structural proteins [13]. The consumption of jaboticaba peel powder (JPP) has been shown to yield antioxidant effects in the liver, kidney, and plasma of obese rats [14] and improve insulin sensitivity in a mouse model of obesity [15]. In addition, JPP treatment has increased fecal triglycerides and attenuated hepatic lipid oxidation but has not protected against hepatic steatosis in obese rats [16]. The treatment with jaboticaba extract has been shown to reduce triacylglycerol and cholesterol plasma levels but not the hepatic lipid peroxidation in a rat model of type 1 diabetes (T1DM) [17]. T1DM model is characterised only by hyperglycaemia but does not mimic the insulin resistance that occurs in T2DM and plays a pivotal role in the disease complications. T2DM responds for 90% of DM cases worldwide; however, JPP effects have not been explored in T2DM models.

This work was aimed to evaluate the effects of dietary supplementation with JPP on the dyslipidemia, oxidative stress, and hepatic damage in a rat model of T2DM.

## 2. Materials and Methods

### 2.1. Jaboticaba Peel Powder

Jaboticaba fruits (*Myrciaria jaboticaba* Vell Berg) were collected, washed, and manually peeled in Campinas (São Paulo, Brazil). The peels were frozen at −18°C and then freeze-dried in a Liobras (São Carlos, São Paulo, Brazil) freeze-dryer to obtain the JPP, which was stored at −80°C until the experimental protocol.

Moisture, ash, and protein content were determined according to [18]. Lipid content was determined according to the Bligh and Dyer method [19]. Total, soluble, and insoluble dietary fibre content were determined according to AOAC [18]. The content of nonfibrous carbohydrate was calculated by subtracting the above percentages from 100%.

The soluble polyphenols of JPP were extracted as described by [20] with some modifications. JPP (1.0 g) was first extracted with 15 mL of a methanol/water/formic acid (85 : 15 : 0.5 v/v) solution, stirred for 30 s in vortex and submitted to ultrasound for 5 min (ULTRA Cleaner 1600, São Paulo, Brazil; 135 W RMS). The extract was centrifuged (2000 ×g, 10 min), and the pellet was exhaustively extracted as described above. Supernatant fractions were combined and used to determine total monomeric anthocyanins by the pH-differential method [21]. Total monomeric anthocyanin content was calculated using the molecular weight (MW) and molar absorptivity (*ε*) of cyanidin-3-glucoside, 449.2 and 26.900, respectively. Results were expressed as cyanidin-3-glucoside equivalents. Supernatant fractions were also used to determine soluble polyphenols [22], using a calibration curve of gallic acid, and total soluble proanthocyanidins using a calibration curve of catechin [23].

After drying at 30°C, the pellet (0.2 g) was treated with 10 mL of butanol/HCl (97.5 : 2.5 v/v) and 0.7 g of FeCl_3_ at 100°C for 1 h. The supernatant obtained was used to determine insoluble polyphenols by measuring the anthocyanidin concentration by assessing the sum of absorbances at 555 and 450 nm and using a calibration curve of a carob pod tannin concentrate [24].

Carotenoids were exhaustively extracted from the JPP (0.5 g) with acetone, transferred to petroleum ether/diethyl ether (1 : 1, v/v), and the solvent was evaporated under N2 flux, and the sample was reconstituted in petroleum ether to assess the total carotenoid concentration using the specific absorption coefficient (A 1 cm 1% = 2396) of *β*-carotene [25].

The composition of soluble polyphenols of the JPP used in this study was assessed by high-performance liquid chromatography coupled to photodiode array and mass spectrometry detectors and comprises 52% anthocyanins and 48% nonanthocyanin phenolics.

### 2.2. Animals and Diabetes Induction

Forty male Wistar rats (eight weeks old, 150–200 g) were supplied by the Central Animal House of the Federal University of Santa Maria (UFSM, RS, Brazil). All procedures were approved by Committee on Care and Use of Experimental Animal Resources/UFSM (protocol no.: 086/2013). Animals were housed in standard polypropylene cages (four rats/cage) and maintained under controlled room temperature (22 ± 2°C) and humidity (55 ± 5%) with 12 : 12 h light and dark cycle with access to feed and water ad libitum.

After a one-week acclimation period, rats were randomly divided in two dietary regimens with access to 30 g diet/day/rat and water ad libitum. The nondiabetic control group received a commercial chow diet (Nuvital CR1, Quimtia, Colombo, Paraná, Brazil), and diabetic groups were fed a high-fat diet (HFD), containing 74% commercial chow (Nuvital CR1, Quimtia, Colombo, Paraná, Brazil), 16% lard, and 10% sucrose (w/w). After 30 days of dietary manipulation, overnight fasted animals were intraperitoneally injected with a freshly prepared solution of streptozotocin (STZ) (1 mL/kg b.w., 35 mg/kg b.w.) in 0.1 M citrate buffer (pH 4.4) [26] or only 0.1 M citrate buffer (1 mL/kg b.w.). Seven days after STZ administration, glycaemia was measured, and only rats that had glycaemia higher than 250 mg/dL were assigned to the diabetic groups.

Rats were then divided into 5 groups (8 animals/group) for an 8-week treatment with JPP as detailed in Table 1. During this period, rats received drinking solutions ad libitum, control rats continued to receive the commercial chow diet (30 g/day/rat), and diabetic rats continued to receive the HFD (30 g/day/rat).

Food and water intake was recorded daily. Body weight was measured every 3 days. At the end of the experiment, rats were fasted for 8 h, and then blood was collected from the caudal vein, and glucose levels were determined with an automatic analyser donated by Roche® of Brazil (Active, Boehringer Mannheim, Indianapolis, Indiana, USA). Subsequently, rats were anaesthetised with isofluorane, and the blood was collected by cardiac puncture into tubes with no additives. Serum was obtained after blood centrifugation at 2000 ×g for 15 min and was then stored at −20°C until biochemical analyses.

A liver portion was homogenised in phosphate buffered saline (PBS) (pH 7.4, 1/8 w/v). Liver homogenate was immediately used to determine thiobarbituric acid reactive substances (TBARS). A fraction of liver homogenate was centrifuged at 2000 ×g for 15 min to yield a supernatant that was used to determine the activity of antioxidant enzymes and the contents of nonprotein thiol groups (NPSH) and protein carbonyl groups.

### 2.3. Serum Biochemical Assays

Insulin was measured in serum by radioimmunoassay using commercial Immunotech kit (Beckman Coulter Company, Marseille, France). The quantitative insulin sensitivity check index (QUICKI) [27] and the fasting insulin resistance index (FIRI) [28] were calculated as follows:

(1) $$\begin{array}{c} \text{QUICKI}=\frac{1}{\left[\log\left(\text{fasting insulin level}\right)+\log\left(\text{fasting glycaemia}\right)\right]},\\ \text{FIRI}=\frac{\left[\text{fasting insulin level}\left(\text{mU}/L\right)+\text{fasting glycaemia}\left(\text{mmol}/L\right)\right]}{25}. \end{array}$$

The serum levels of total cholesterol (TC), triglycerides (TG), low-density lipoprotein (LDL) and high-density lipoprotein (HDL) cholesterol, glucose, and the activities of aspartate aminotransferase (ALT) and alanine aminotransferase (AST) were determined by enzymatic methods, using commercial kits (Doles, Goiania, GO, Brazil). The serum level of very-low-density lipoprotein cholesterol (VLDL) was calculated by Friedewald's equation [29]:

(2) $$\begin{array}{c} \text{VLDL}=\frac{\text{TG}}{5}. \end{array}$$

The atherogenic index of plasma (AIP) was calculated conforming to [30] with the formula

(3) $$\begin{array}{c} \text{AIP}=\log\left(\frac{\text{TG}}{\text{HDL}}\right). \end{array}$$

### 2.4. Markers of Oxidative Stress

Lipid peroxidation in liver homogenate was estimated colorimetrically by measuring TBARS [31]. Hepatic protein carbonyl group was determined by reaction with 2,4-dinitrophenylhydrazine (DNPH) at 240 nm [32]. Hepatic NPSH levels were measured as described in [33] after sample deproteinization with 4% trichloroacetic acid. The levels of reduced glutathione (GSH) and oxidised glutathione (GSSG) were measured by the fluorimetric method using O-phthalaldehyde [34], and results were expressed as the GSH/GSSG ratio.

### 2.5. Antioxidant Enzymes Activities

Superoxide dismutase (SOD) activity was determined at 480 nm [35]. Catalase (CAT) activity was measured at 240 nm [36]. Thioredoxin reductase-1 (TrxR-1) activity was determined at 412 nm [37].

Glutathione peroxidase (GPx) activity was determined at 340 nm [38]. Glutathione reductase (GR) activity was determined according to [39]. Glutathione *S*-transferase (GST) activity was determined at 340 nm [40].

Hepatic *δ*-aminolaevulinic acid dehydratase (*δ*-ALA-D) activity was assayed by measuring the rate of porphobilinogen (PBG) formation using Ehrlich's reagent at 555 nm after sample incubation for 1 h at 37°C [41].

The protein content of liver tissue supernatant was measured using bovine serum albumin as standard [42] and used to calculate enzyme activities in liver tissue.

### 2.6. Inflammatory Cytokine Level

TNF-*α* concentration in serum samples was analysed by ELISA (eBIOSCIENCE®, San Diego, CA, USA).

### 2.7. Quantitative Real-Time RT-PCR (qRT-PCR) Analysis

The gene expression of enzymes involved in the glutathione synthesis and apoptosis markers were assessed in liver samples. The RNA extraction was performed using Trizol reagent following the manufacturer's instructions (Ludwig-Biotec, São Paulo, Brazil). The RNA extracted was measured by a Thermo Scientific NanoDropTM 1000 spectrophotometer. To perform reverse transcription, RNA was added to the samples of RNA (1000 ng/*μ*l) with 0.2 *μ*l of DNAase (Invitrogen Life Technologies, Carlsbad, CA, USA) at 37°C for 5 minutes followed by heating at 65°C for 10 minutes. The cDNA was generated with Iscript cDNA and Mix Iscript (Bio-Rad Laboratories, Hercules, CA, USA). qRT-PCR was conducted in the Rotor-Gene Q 5plex HRM System (Qiagen Biotechnology, Germany) with 2x QuantiFast SYBR Green PCR Master Mix (Qiagen Biotechnology, Germany). qRT-PCR reactions were run in triplicate, using 1 *µ*M of each primer, 1000 ng/*µ*L of cDNA, RNAase-free water, and 2x QuantiFast SYBR® Green PCR Master Mix (Qiagen Biotechnology, Germany). *β*-Actin was used as the housekeeping gene, and its expression level was used as an internal control. The relative gene expression was calculated using the comparative cytosine-thymine (Ct) method and was expressed as the fold expression compared to the control. The specific primer pairs used are described in Table 2.

### 2.8. Statistical Analyses

Data were analysed using one-way analysis of variance (ANOVA) followed by post hoc Duncan's multiple range test or Dunnett's test (for gene expression data), when necessary. Data that did not meet the ANOVA assumptions were submitted to the nonparametric Kruskal–Wallis analysis, followed by a multiple comparison test. Results were expressed as the mean ± SEM, and differences were considered statistically significant when *p* < 0.05. Data were analysed using the Statistica® 9.1 software system (Statsoft Inc., 2004).

## 3. Results

JPP contains important phytochemicals such as polyphenols and carotenoids (Table 3). Among these phytochemicals, soluble polyphenols are found at the greatest concentration, being composed mostly by cyanidin-3-glucoside, ellagitanins and gallotanins (Table 4). JPP is also a good source of insoluble polyphenols, as well as carotenoids. Besides, JPP has considerable amount of total dietary fibre (24.4%), being 9.3% soluble dietary fibre (Table 3).

The average drinking intake for diabetic rats amounted to 265 mL/day/kg b.w. and did not differ among all diabetic groups during the treatment (data not shown).

Before treatment with JPP, rats that received high-fat diet and low doses of STZ to induce T2DM had increased glycaemia when compared to the control group (Table 5; *p* < 0.05). After 8-week treatment, only rats that received the highest dose of JPP (JPP-III) had lower glycaemia (19%) than the diabetic-vehicle group. T2DM induction did not alter fasting serum insulin levels. However, the diabetic JPP-I group had lower insulin level than the diabetic-vehicle group (Table 5; *p* < 0.05). T2DM induction increased insulin resistance (FIRI index) and decreased insulin sensitivity (QUICKI index) when compared to the control group (Table 5; *p* < 0.05). JPP treatment reduced to control levels the insulin resistance and improved insulin sensitivity only at the lowest dose (JPP-I; Table 5; *p* < 0.05).

**Figure 1:**
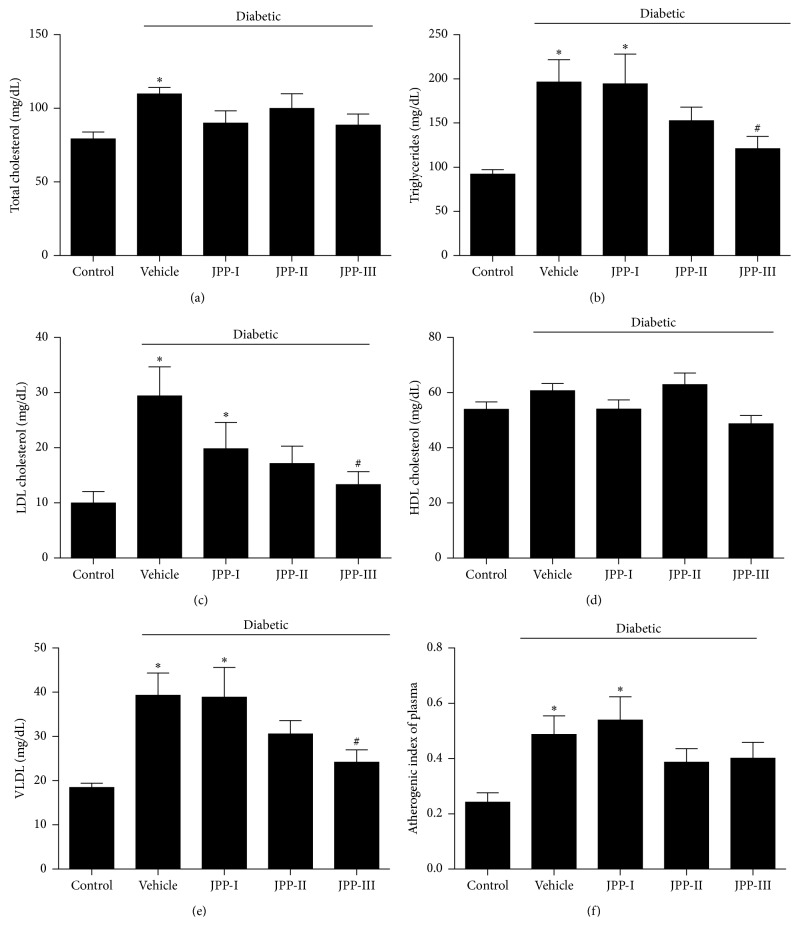
Serum levels of total cholesterol (a), triglycerides (b), LDL (c), HDL (d), and VLDL (e) and the atherogenic index (f) of diabetic rats fed high-fat diet and treated with JPP or vehicle for 8 weeks (means ± SEM, *n*=8). ^*∗*^Different from the control group (*p* < 0.05); ^#^different from the diabetic-vehicle group (triglycerides, cholesterol, and VLDL: ANOVA-Duncan's test and HDL and LDL: Kruskal–Wallis multiple comparison test *p* < 0.05); JPP: Jaboticaba peel powder; VLDL: very low-density lipoprotein; LDL: low-density lipoprotein; HDL: high-density lipoprotein.

T2DM induction increased the levels of TC, TG, LDL-cholesterol and VLDL-cholesterol, and the atherogenic index in comparison to the control group (Figures 1(a)–1(c), 1(f), and 1(e); *p* < 0.05), whereas the HDL-cholesterol levels did not differ among groups (Figure 1(d); *p* > 0.05). JPP treatment did not affect HDL-cholesterol levels (Figure 1(d)) but reduced the total cholesterol levels in diabetic rats at all doses tested (Figure 1(a); *p* < 0.05). In addition, treatment with JPP-II and JPP-III also reduced TG, LDL-cholesterol, and VLDL-cholesterol levels (Figures 1(b), 1(c), and 1(e); *p* < 0.05) and attenuated the cardiovascular risk (AIP, Figure 1(f); *p* < 0.05) to values similar to the control levels but JPP-I had no effect.

**Figure 2:**
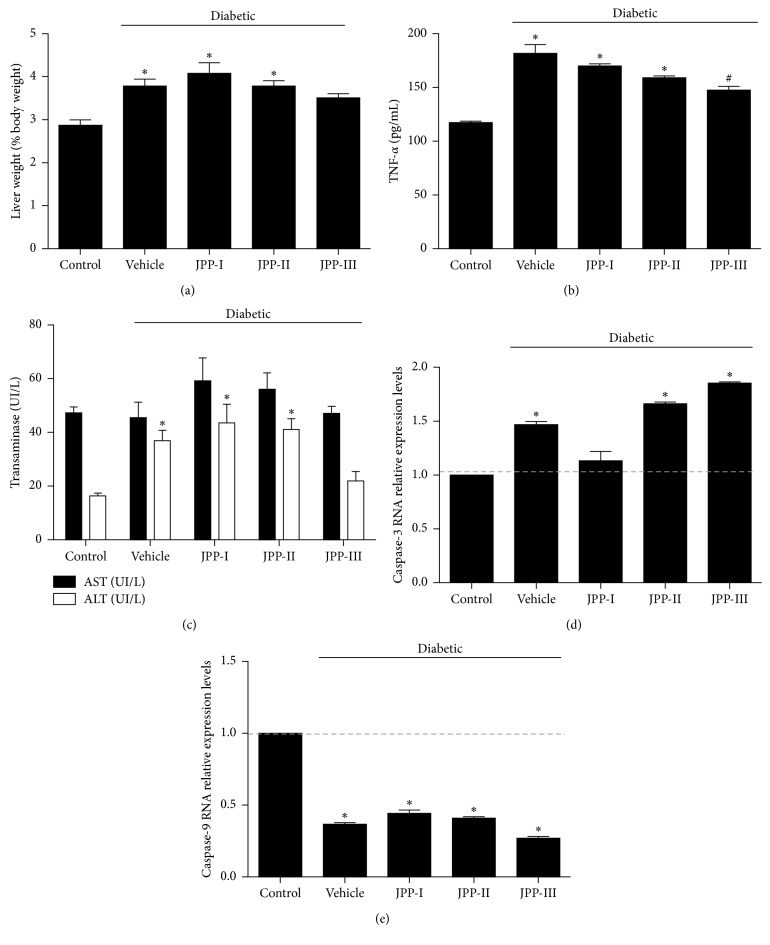
Liver weight (a), serum level of TNF-*α* (b), serum transaminases activity (c), hepatic expression of caspase-3 (d), and hepatic expression of caspase-9 (e) in diabetic rats fed high-fat diet and treated with JPP or vehicle for 8 weeks (means ± SEM, *n*=8). ^*∗*^Different from the control group. ^#^Different from the diabetic-vehicle group (ALT, liver histology: ANOVA-Duncan's test; liver weight: Kruskal–Wallis multiple comparison test; caspase-3 and caspase-9: ANOVA-Dunnett's test; *p* < 0.05). The gene expression data were normalized by *β*-Actin gene. Mean ± SD were obtained from three independent repetitions. ALT: aspartate aminotransferase; AST: alanine aminotransferase; JPP: jaboticaba peel powder; TNF-*α*: tumour necrosis factor alfa.

The diabetic-vehicle group had higher liver weight (31.7%) and serum levels of the inflammatory marker TNF-*α* (54.9%) and ALT activity (125.2%) than the control group (Figures 2(a)–2(c); *p* < 0.05), although AST serum activity did not differ among groups (Figure 2(c), *p* > 0.05). JPP treatment at the highest dose (JPP-III) prevented the increase of liver weight (Figure 2(a)), TNF-*α* levels (Figure 2(b)), and ALT activity (Figure 2(c)) in diabetic rats (*p* < 0.05).

Hepatic gene expression of caspase-3 increased after induction of T2DM when compared to control group, whereas caspase-9 gene expression decreased with induction of T2DM (Figures 2(d) and 2(e); *p* < 0.05). Only JPP-I treatment was able to prevent the increase in caspase-3 expression (Figure 2(d); *p* < 0.05), but no treatment was able to restore caspase-9 gene expression (Figure 2(e); *p* > 0.05).

**Figure 3:**
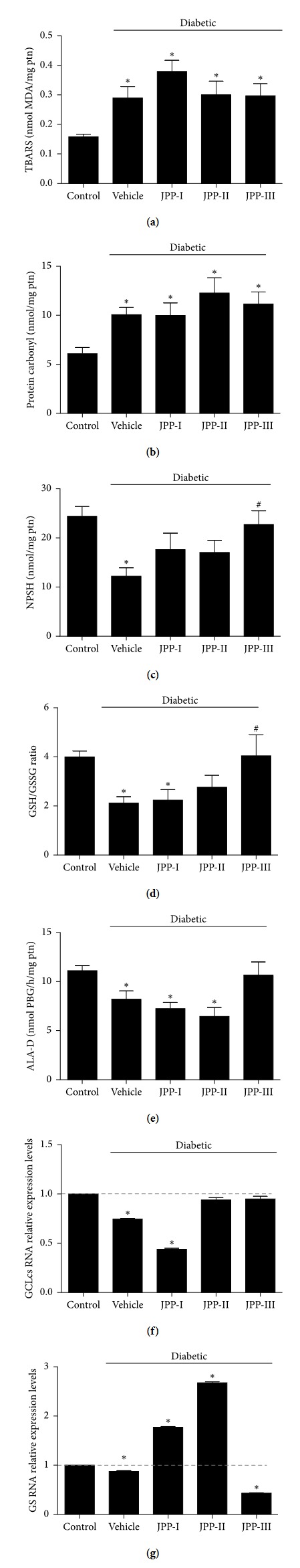
TBARS (a), protein carbonyl (b) and NPSH (c) levels, GSH/GSSG ratio (d), *δ*-ALA-D activity (e), and gene expression of GCLcs (f) and GS (g) in the liver of diabetic rats fed high-fat diet and treated with jaboticaba peel powder or vehicle for 8 weeks (means ± SEM, *n*=8). ^*∗*^Different from the control group (*p* < 0.05). ^#^Different from the diabetic-vehicle group (TBARS, protein carbonyl, NPSH, GSH/GSSG ratio, and *δ*-ALA-D: ANOVA-Duncan's test; GCLcs and GS: ANOVA-Dunnett's test; *p* < 0.05). The gene expression data were normalized by *β*-actin gene expression, and mean ± SD were obtained from three independent repetitions. TBARS = thiobarbituric acid reactive substances; NPSH = nonprotein thiol group; GSH = reduced glutathione; GSSG = oxidised glutathione; *δ*-ALA-D = *δ*-aminolaevulinic acid dehydratase; GCLcs = glutamate-cysteine ligase, catalytic subunit; GS = glutathione synthase.

T2DM induction increased hepatic lipid oxidation assessed by TBARS levels (82.9%) and protein oxidation assessed by the content of protein carbonyl groups (65.4%) in comparison to the control group (Figures 3(a) and 3(b); *p* < 0.05). JPP treatment did not prevent the increase in lipid or protein oxidation (Figures 3(a) and 3(b); *p* > 0.05).

T2DM induction also reduced NPSH levels (49.8%), the GSH/GSSG ratio (47.0%), and the activity of the sulfhydryl-containing enzyme *δ*-ALA-D (26.1%) in liver compared to the control group (Figures 3(c)–3(e); *p* < 0.05). After 8 weeks of JPP treatment, the depletion of NPSH groups in liver was partially prevented by JPP-I and JPP-II, whereas JPP-III completely restored NPSH levels (Figure 3(c); *p* < 0.05). The GSH/GSSG ratio was partially recovered by JPP-II and completely recovered by JPP-III supplementation (Figure 3(d); *p* < 0.05). ALA-D activity was also recovered by JPP treatment but only at the highest dose (Figure 3(e); *p* < 0.05).

Additionally, T2DM reduced gene expression of GCLcs (25.3%) and GS (12.3%), two enzymes involved in GSH synthesis, in comparison to the control group (Figures 3(f) and 3(g); *p* < 0.05). The treatment with JPP-II and JPP-III recovered gene expression of GCLcs up to control level (Figure 3(f); *p* < 0.05). On the other hand, the treatment with JPP-I and JPP-II increased gene expression of GS expression above control levels (*p* < 0.05), but the treatment with JPP-III failed to do so (Figure 3(g); *p* < 0.05).

T2DM reduced the activities of hepatic antioxidant enzymes SOD (31.2%), CAT (48.5%), TrxR-1 (48.2%), GPx (57.5%), and GST (20.6%) but not GR when compared to the control group (Table 6, *p* < 0.05). JPP-III supplementation decreased GR activity (Table 6, *p* < 0.05). No JPP doses restored SOD, CAT, TrxR-1, GPx, or GST activities impaired by T2DM (Table 6, *p* > 0.05).

## 4. Discussion

Currently much attention has been focused on the potential of fruit phytochemicals, especially polyphenols, to prevent and treat chronic diseases such as T2DM [43]. Extractable polyphenols and anthocyanins were the main bioactive compounds found in JPP, and they have been associated to the improvement of oxidative stress, insulin resistance, and lipid profile in a rat model of obesity [14, 15, 44].

The content of JPP's insoluble polyphenols, which refers to the polyphenols bound to fibres and protein, was higher than the amount found in other freeze-dried fruits, as cherry, white grape, strawberry, and apple with peel [45]. Insoluble polyphenols can be hydrolysed by gut microbiota yielding phenolic metabolites that can be absorbed promoting beneficial health effects [46]. Additionally, the JPP used in this study had higher content of carotenoids (1.78 mg/100 g JPP) than the jaboticaba fruit of the same species cultivated in Minas Gerais (Brazil) (0.87 mg *β*-carotene/100 g dry weight basis) [13].

We have demonstrated that the consumption of JPP for 8 weeks reduced glycaemia, dyslipidemia, and hepatic complications in a rat model of T2DM, providing evidence that JPP treatment has beneficial protective effects even after diabetes and insulin resistance are installed. Distinct mechanisms seem to underline the protective effects of JPP at the different doses tested. While the highest JPP dose decreased end glycaemia, the lowest JPP dose decreased insulin levels. Most protective effects of JPP had a linear dose-response behaviour and were more evident at the highest dose of JPP, namely, the decrease of liver hypertrophy and plasma alanine aminotransferase activity (a marker of liver damage) and the increase of liver thiol levels (increased NPSH levels, GSH/GSSG ratio) and ALA-D activity. Similar dose-response behaviour was observed for the protective effects of JPP against diabetes-induced dyslipidemia. On the other hand, the beneficial effects of JPP by improving insulin signalling and decreasing the expression of caspase-3 did not obey a linear dose-response behaviour as they were observed only at the lowest dose of JPP. These effects seem to be better explained by a hormetic response [47].

Anthocyanins likely contributed to the antidiabetic effect of JPP as the intake of cyanidin 3-glucoside either purified or from Queen Garnet plum juice (7.4–7.6 mg anthocyanin/kg b.w./day) has been shown to decrease insulinemia and improve glucose tolerance in diet-induced metabolic syndrome in rats [48]. In the present study, we found similar results after the intake of JPP-I (daily average intake of 10.3 mg anthocyanins/kg b.w.). This lowest dose of JPP improved insulin signalling as indicated by the increased insulin sensitivity (QUICKI) and decreased insulin resistance (FIRI). These effects were caused by the ability of JPP-I to remarkably reduce serum insulin levels, while keeping glycaemia levels similar to the vehicle group. The decrease of insulin levels triggered by JPP disappeared at the highest JPP doses indicating a U-shaped effect characteristic of hormetic responses [47]. This biphasic dose response model has been shown to be better than the linearity and threshold-response models to explain the behaviour of various toxic and therapeutic drugs [49] and appears to explain the effect of JPP on insulin signalling. On the other hand, the hypoglycaemic effect of JPP exhibits a linear dose-response behaviour (observed at the highest dose, JPP-III; daily average intake of 37.5 mg anthocyanins/kg b.w.). This effect may be related to low intestinal glucose absorption either due to dietary fibre or due to the inhibition of digestive *α*-amylase and *α*-glucosidase activities by phenolic compounds as recently reported for jaboticaba extracts [50].

Dyslipidemia can result in cardiovascular complications in T2DM patients [9], and the atherogenic index can predict future cardiovascular disease even before the development of diabetes (prediabetes) [51]. Accordingly, we found an increased atherogenic index in the diabetic-vehicle group. JPP treatment improved the lipid profile, mainly by decreasing triglycerides and LDL levels, which consequently attenuated the atherogenic index. Soluble dietary fibre probably contributed to the lipid-lowering effect of JPP, because it increases the viscosity of the intestinal content and limits fat absorption by impeding the action of bile salts and enzymes or by sequestering bile salts [52]. Accordingly, the hypocholesterolemic and hypotriglyceridemic effects of riceberry in a rat model of T2DM have been attributed to the presence of fibre (soluble and insoluble) and not to the polyphenols [5].

The consumption of fat diet along with the insulin resistance can further increase circulating FFA, which promotes lipotoxicity by increasing VLDL and triglycerides synthesis in hepatic tissue. Thus, fat liver accumulation in nontreated T2DM patients can result in hepatic steatosis or nonalcoholic fatty liver disease (NAFLD), when fat accumulation is associated with inflammatory process [4]. Accordingly, the rat model of T2DM exhibited dyslipidemia, insulin resistance, inflammatory response (TNF-*α*), and hepatic damage indicated by increased activity of serum transaminases and liver hypertrophy and apoptosis (increased gene expression of caspase-3). JPP-III treatment was able to improve the dyslipidemia, most likely by reducing lipid accumulation in the liver of T2DM rats, which may be associated with the decrease of liver weight. JPP-III treatment attenuated hepatic damage as indicated by the reduced serum ALT activity and liver hypertrophy. These protective effects of JPP were not associated to the prevention of hepatic lipid oxidation or apoptosis. Excess glucose promotes an imbalance between reactive species and antioxidant defences being responsibly for tissue damage in the T2DM [52]. Accordingly, we found increased lipid and protein oxidation with concomitant reduction of enzymatic antioxidant defences (SOD, CAT, TrxR-1, GPx, and GST activities) in the liver of diabetic rats. T2DM induction also depleted nonenzymatic antioxidant defences assessed by the hepatic content of NPSH and the GSH/GSSG ratio. GSH, which is the major contributor to the hepatic content of NPSH, can modulate cell death by regulating the redox state of specific thiol residues of proteins, such as caspases [54]. The depletion of NPSH levels and the decrease of GSH/GSSG ratio in T2DM rats were not associated to an increase in GST activity, which uses GSH to detoxify xenobiotics. Also the depletion of GSH was not associated to an increase in GPx activity, which uses GSH to remove peroxides, or to a decreased GR activity, which reduces oxidised GSH. GSH depletion could be possibly associated to a failure in the conversion of the oxidised gluthatione into GSH by the thioredoxin protein or thioredoxin system [55], confirmed by the decrease in TrxR-1 activity with T2DM induction. We observed that the depletion of NPSH and GSH (GSH/GSSG ratio) content was accompanied by decreased activity of *δ*-ALA-D. The sulfhydryl-containing enzyme *δ*-ALA-D was inhibited upon oxidation of its sulfhydryl groups and has been suggested to be a sensor for oxidative stress [56]. Thus, GSH depletion in diabetic rats was functionally relevant and indicates an imbalance in the thiol/disulfide redox state that probably contributed to the hepatic oxidative stress and tissue damage.

Despite restoring GSH levels (NPSH and GSH/GSSG ratio) and the activity of *δ*-ALA-D in diabetic rats, JPP treatment was not able to decrease the hepatic oxidative damage in lipids or protein oxidation assessed by protein carbonylation. The improvement of NPSH levels and GSH/GSSG ratio caused by JPP-III was most likely caused by increased GSH synthesis due to recovered gene expression of GCLcs, which is the rate-limiting step in GSH biosynthesis [54]. Cyanidin-3-glucoside has been shown to increase hepatic GSH synthesis in a culture of liver human cells (HepG2) by inducing GCLcs expression via the PKA–CREB signalling pathway [57]. The decrease in the expression of GS observed in the present study for the JPP-III group (0.4 times that of control and vehicle group) will not be expected to decrease GSH synthesis because the activity of GS, which is upstream GCL in glutathione synthesis, is normally 2 to 4 times higher than GCL [54]. Accordingly, NPSH levels and GSH/GSSG ratio were linearly increased with the increase in JPP dose as observed for the gene expression of GCL.

The beneficial effect of JPP on *δ*-ALA-D activity is very important, since this is the rate-limiting enzyme for the biosynthesis of heme, the prostetic group of haemoglobin. We can speculate that the protective effect of JPP occurred by preventing the oxidation of sulfhydryl groups in the active site of *δ*-ALA-D. This antioxidant mechanism is likely caused by the increased synthesis of GSH due to the upregulation of gene expression of GCLcs, the main regulatory enzyme for glutathione biosynthesis in liver.

Liver complications were observed in T2DM rats as indicated by the tissue hypertrophy and increased activity of ALT in the serum, which is a better marker of liver damage than AST activity that remained unchanged. ALT activity has been long considered as a specific indicator of hepatic damage, because it is found at higher concentration in liver than in other tissues [58]. Lipid and protein oxidation reveals hepatic oxidative stress in our T2DM model, which has been shown to alter transport function and membrane permeability in hepatocytes [53] and could contribute to the increase in serum transaminase [59]. The hepatic damage was confirmed by the activation of the apoptotic pathway as we observed an increase in gene expression of caspase-3 in the diabetic-vehicle group. Caspase-3 was most likely activated by the extrinsic pathway (caspases 8 and 10) as the gene expression of caspase 9, which belongs to the intrinsic pathway, was indeed reduced in diabetic rats. Additionally, a decrease in the redox status of GSH and thioredoxin protein (Trx) can induce JNK-dependent apoptosis [61]. The oxidation of Trx triggers signalling for the expression of proapoptotic factors, such as TNF-*α*, FasL, and Bak [60]. In addition, apoptosis can be activated by a caspase-independent pathway when the GSH levels decline, via activation of the apoptosis inducing factor (AIF) that causes direct DNA fragmentation [61]. Hyperglycaemia has been shown to increase the intrinsic apoptosis pathway/mitochondrial through oxidative stress and Bax protein expression in diabetes models [62]. However, our T2DM model was characterised by increased dietary fat content and inflammatory process (elevated TNF-*α* levels), which can activate the extrinsic apoptosis. In fact, the activation of TRL-4 and TNF-*α* receptors in liver has been shown to activate the extrinsic apoptosis pathway with activation of caspase 8 and 10 in a rat model of T1DM [63].

Liver necrosis can occur simultaneously with apoptosis, and both mechanisms of cell death involve caspase activation. Necrosis is a mechanism of unregulated cell death, which involves an exacerbated inflammatory process and the loss of cellular permeability [64]. The rupture of cellular membrane releases cytosolic transaminases into serum and triggers cellular events causing cell swelling. The hepatic accumulation of triglycerides together with the activation of cell death by necrosis can contribute to the hepatic hypertrophy in T2DM rats. Thus, our T2DM model was likely associated to necrosis as indicated by the release of ALT into serum, the inflammatory process, and the increased gene expression of caspase-3.

Although JPP treatment at the highest doses (II and III) was not able to decrease caspase-3 expression, the consumption of JPP at the highest dose promoted an initial recovery of hepatic damage by attenuating the inflammatory response and preventing changes in liver weight and serum ALT activity, which is likely related to the preservation of hepatocyte cell membrane integrity. On the other hand, only the lowest dose of JPP was able to decrease gene expression of caspase-3. The decrease of caspase-3 expression in JPP-I may be explained by the reduction in insulin levels, since insulin can induce apoptosis by activating caspase-3 via phosphatidylinositol 3-kinase (PI3-kinase) pathway [65].

Additionally, JPP treatment recovered T2DM dyslipidemia in a dose-dependent manner and restored nonenzymatic antioxidant defences (NPSH and GSH/GSSG ratio), without changes in the enzymatic antioxidant defences (SOD, CAT, TrxR-1, GPx, and GST activities). The highest dose of JPP was particularly effective for recovering the sulfhydryl reducing status of liver tissue as it yielded the greatest increase in NPSH levels, GSH/GSSG ratio, and prevented *δ*-ALA-D inhibition. Thus, our data suggest that the modulation of thiol/disulfide redox state, rather than modulation of the apoptosis pathway, is the major mechanism responsible for the protective effect of JPP against hepatic damage in T2DM.

Our data indicate that JPP had a relevant protective role by preserving thiol/disulfide redox balance and protecting sensitive protein thiols from irreversible oxidation triggered by diabetes-induced oxidative conditions. During oxidative stress, protein cysteine residues (Prot-SH) can be oxidised to sulfenic acid (Prot-SOH), which can react with GSH to form protein mixed disulfides Prot-SDG (glutathionylation), which in turn can be reduced back to Prot-SH via glutaredoxin (Grx) or sulfiredoxin (Srx) [54]. This is a mechanism to protect sensitive protein thiols from irreversible oxidation [54].

In conclusion, the present study showed that JPP treatment at the highest dose (JPP-III) attenuated hyperglycaemia, insulin resistance, hyperlipidemia and hepatic complications in T2DM model. Hepatic protection was likely mediated by the increase in GSH synthesis that restored NPSH levels and probably prevented the loss of activity of sulfhydryl-containing enzymes.

## Figures and Tables

**Table 1:** Treatment of experimental groups.

Groups	Diet	Injection (i.p.)	Drinking solution
Control (nondiabetic)	Commercial chow diet	Vehicle^*∗*^	Vehicle^#^
Diabetic-vehicle	HFD	STZ	Vehicle^#^
Diabetic JPP-I	HFD	STZ	2.7 g JPP/L of vehicle
Diabetic JPP-II	HFD	STZ	5.4 g JPP/L of vehicle
Diabetic JPP-III	HFD	STZ	10.8 g JPP/L of vehicle

^*∗*^STZ vehicle was 0.1 M citrate buffer, pH 4.4. ^#^Water containing 0.5% carboxymethyl cellulose was used as vehicle to stabilize the drinking suspension of JPP. STZ: streptozotocin; HFD: high-fat diet.

**Table 2:** Primer sequences.

Primers	Forward	Reverse
Caspase-3	GAGACAGACAGTGGAACTGACGATG	GGCGCAAAGTGACTGGATGA
Caspase-9	CTGAGCCAGATGCTGTCCCATA	GACACCATCCAAGGTCTCGATGTA
Glutamate-cysteine ligase, catalytic subunit (GCLcs)	GTGGACACCCGATGCAGTAT	TCATCCACCTGGCAACAGTC
Glutathione synthase (GS)	GCAGGAACTGAGCAGGGTG	GCTTCAGCACAAAGTGGCTAG
*β*-Actin	GCAGGAGTACGATGAGTCCG	ACGCAGCTCAGTAACAGTCC

**Table 3:** Composition of freeze-dried jaboticaba peel powder.

	Amount (mean ± SD)
*Proximate composition*	
Moisture (%)	17.1 ± 0.2
Ash (%)	3.3 ± 1.0
Protein (%)	5.6 ± 0.0
Lipids (%)	1.3 ± 0.2
Total dietary fibre (%)	24.4 ± 1.0
Soluble dietary fibre (%)	9.3 ± 0.9
Insoluble dietary fibre (%)	15.1 ± 0.1
Nonfibrous carbohydrates (%)	48.3 ± 0.4

*Phytochemicals*	
Soluble polyphenols (g gallic acid equivalents/100 g JPP)	9.67 ± 0.42
Insoluble polyphenols (g condensed tannins/100 g JPP)	0.73 ± 0.15
Carotenoids (mg *β*-carotene equivalents/100 g JPP)	1.78 ± 0.13

**Table 4:** Composition of soluble polyphenols of freeze-dried jaboticaba peel powder evaluated by LC-PDA-MS^*n*^.

	Amount (%)
*Anthocyanins*	*52*
Cyanidin-3-glucoside	90.15
Delphinidin-3-glucoside	9.38
Pelargonidin-3-glucoside	0.17
Peonidin-3-glucoside	0.31

*Nonanthocyanin phenolics*	*48*
Ellagitannins and Gallotannins	69.57
Quercetin-hexoside	2.35
Ellagic acid	4.95
Myricetin-rhamnoside	1.44

**Table 5:** Effect of JPP treatment on the glycaemia, insulinemia, insulin sensitivity, and insulin resistance of control and diabetic rats.

	Control	Diabetic			
		Vehicle	JPP-I	JPP-II	JPP-III
Onset glucose (mg/dL)	102.3 ± 1.5	353.3 ± 29.9^*∗*^	366.7 ± 20.6^*∗*^	362.0 ± 16.7^*∗*^	369.7 ± 42.2^*∗*^
End glucose (mg/dL)	96.7 ± 1.9	469.0 ± 15.6^*∗*^	443.3 ± 20.1^*∗*^	422.5 ± 15.1^*∗*^	379.5 ± 36.4^*∗*^^#^
Insulin (mU/L)	5.12 ± 0.6	7.39 ± 1.4	3.64 ± 0.7^#^	10.10 ± 2.7	8.45 ± 2.5
QUICKI	0.71 ± 0.02	0.46 ± 0.02^*∗*^	0.53 ± 0.02^*∗*^^#^	0.44 ± 0.02^*∗*^	0.49 ± 0.03^*∗*^
FIRI	0.56 ± 0.05	0.82 ± 0.09^*∗*^	0.58 ± 0.06^#^	0.86 ± 0.08^*∗*^	0.79 ± 0.08^*∗*^

Glycaemia was assessed before (onset) and after (end) JPP treatment, whereas the other measures were only made after JPP treatment. Data are presented as means ± SEM (*n*=8). ^*∗*^Different from the control group. ^#^Different from the diabetic-vehicle group (ANOVA followed by Duncan's test, *p* < 0.05). QUICKI: quantitative insulin sensitivity check index; FIRI: fasting insulin resistance index.

**Table 6:** Effect of JPP treatment on the activity of antioxidant enzymes in the liver of control and diabetic rats.

	Control	Diabetic			
		Vehicle	JPP-I	JPP-II	JPP-III
SOD (U/mg ptn)	27.2 ± 0.7	18.7 ± 0.7^*∗*^	21.0 ± 1.3^*∗*^	19.3 ± 0.9^*∗*^	20.4 ± 1.7^*∗*^
CAT (K/mg ptn)	32.6 ± 1.8	16.8 ± 0.9^*∗*^	17.3 ± 0.9^*∗*^	19.2 ± 1.0^*∗*^	20.1 ± 3.3^*∗*^
TrxR-1 (nmol DTNB/min/mg ptn)	14.1 ± 0.6	7.3 ± 0.7^*∗*^	9.8 ± 0.9^*∗*^	8.8 ± 0.9^*∗*^	9.0 ± 1.1^*∗*^
GPx (nmol NADPH/min/mg ptn)	4.0 ± 0.8	1.7 ± 0.6^*∗*^	1.1 ± 0.3^*∗*^	1.1 ± 0.2^*∗*^	1.9 ± 0.4^*∗*^
GR (nmol NADPH/min/mg ptn)	13.3 ± 1.1	9.6 ± 2.4	9.9 ± 0.7	13.3 ± 1.6	3.8 ± 0.7^*∗*^^#^
GST (nmol CDNB/min/mg ptn)	504.1 ± 13.6	400.3 ± 28.4^*∗*^	344.5 ± 16.7^*∗*^	377.7 ± 22.8^*∗*^	356.3 ± 44.3^*∗*^

Data are presented as means ± SEM (*n*=8); ^*∗*^different from the control group (CAT, SOD, and TrxR-1: Kruskal–Wallis multiple comparison test and GST, GPx, and GR: ANOVA-Duncan's test, *p* < 0.05); JPP: jaboticaba peel powder; SOD: superoxide dismutase; CAT: catalase; TrxR-1: thioredoxin reductase-1; GPx: glutathione peroxidase; GR: glutathione reductase; GST: glutathione *S*-transferase; ptn: protein; DTNB: 5,5′-ditiobis(2-nitrobenzoic acid); NADPH: nicotinamide adenine dinucleotide phosphate reduced; CDNB: 1-chloro-2,4-dinitrobenzene.

## Data Availability

The authors will provide the data related to the results contained in this article when necessary.
